# Supplementary material for: Genome Survey of Male and Female Spotted Scat (Scatophagus argus)
Source: Animals (Basel). 2019 Dec 11;9(12):1117. doi: 10.3390/ani9121117 (PMC6940847; doi:10.3390/ani9121117)
Supplement: Supplementary file 1 [file animals-09-01117-s001.pdf]

Supplementary Figures

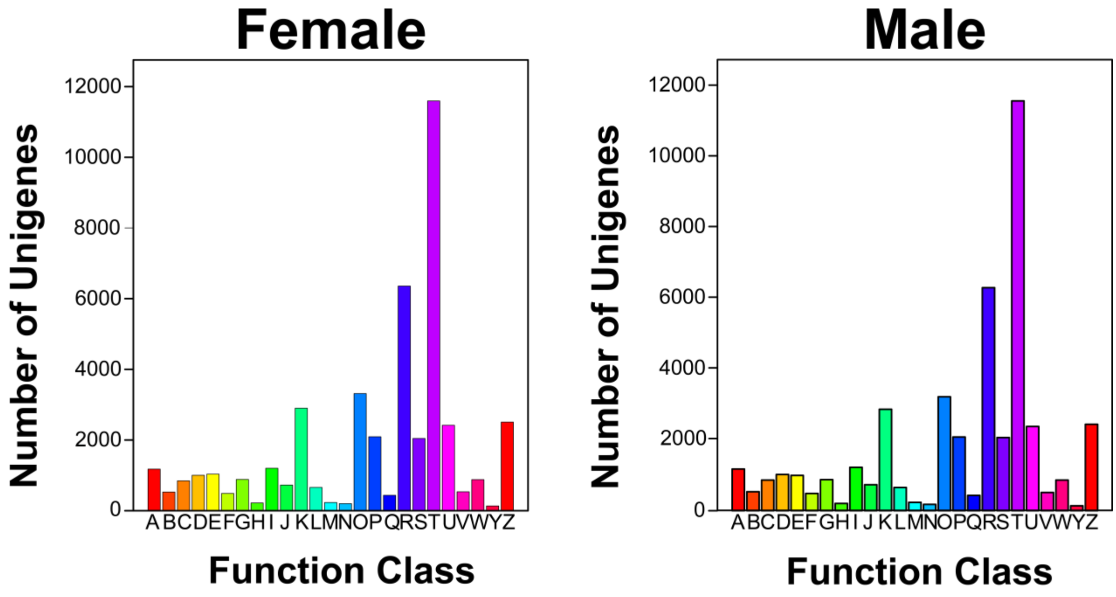

Figure. S1. KOG function classification of predicted genes in *S. argus.* The different color code (A–Z) represented different category as listed at right of the histogram. A: RNA processing and modification. B: Chromatin structure and dynamics. C: Energy production and conversion. D: Cell cycle control, cell division, chromosome partitioning. E: Amino acid transport and metabolism. F: Nucleotide transport and metabolism. G: Carbohydrate transport and metabolism. H: Coenzyme transport and metabolism. I: Lipid transport and metabolism. J: Translation, ribosomal structure and biogenesis. K: Transcription. L: Replication, recombination and repair. M: Cell wall/membrane/envelope biogenesis. N: Cell motility. O: Posttranslational modification, protein turnover, chaperones. P: Inorganic ion transport and metabolism. Q: Secondary metabolites biosynthesis, transport and catabolism. R: General function prediction only. S: Function unknown. T: Signal transduction mechanisms. U: Intracellular trafficking, secretion, and vesicular transport. V: Defense mechanisms. W: Extracellular structures. Y: Nuclear structure. Z: Cytoskeleton.

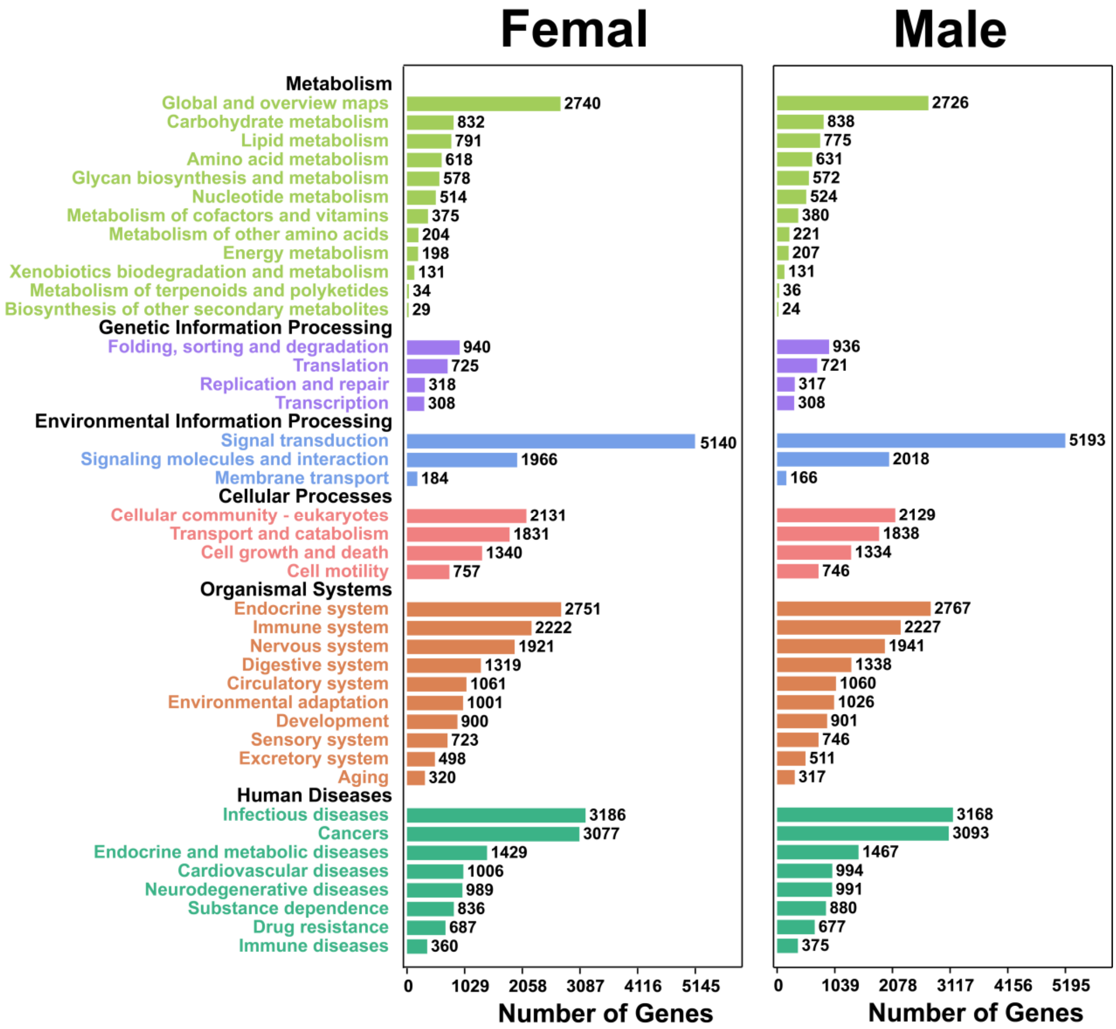

Figure. S2. KEGG pathway annotation of predicted genes in *S. argus*. The different color code represented different category.

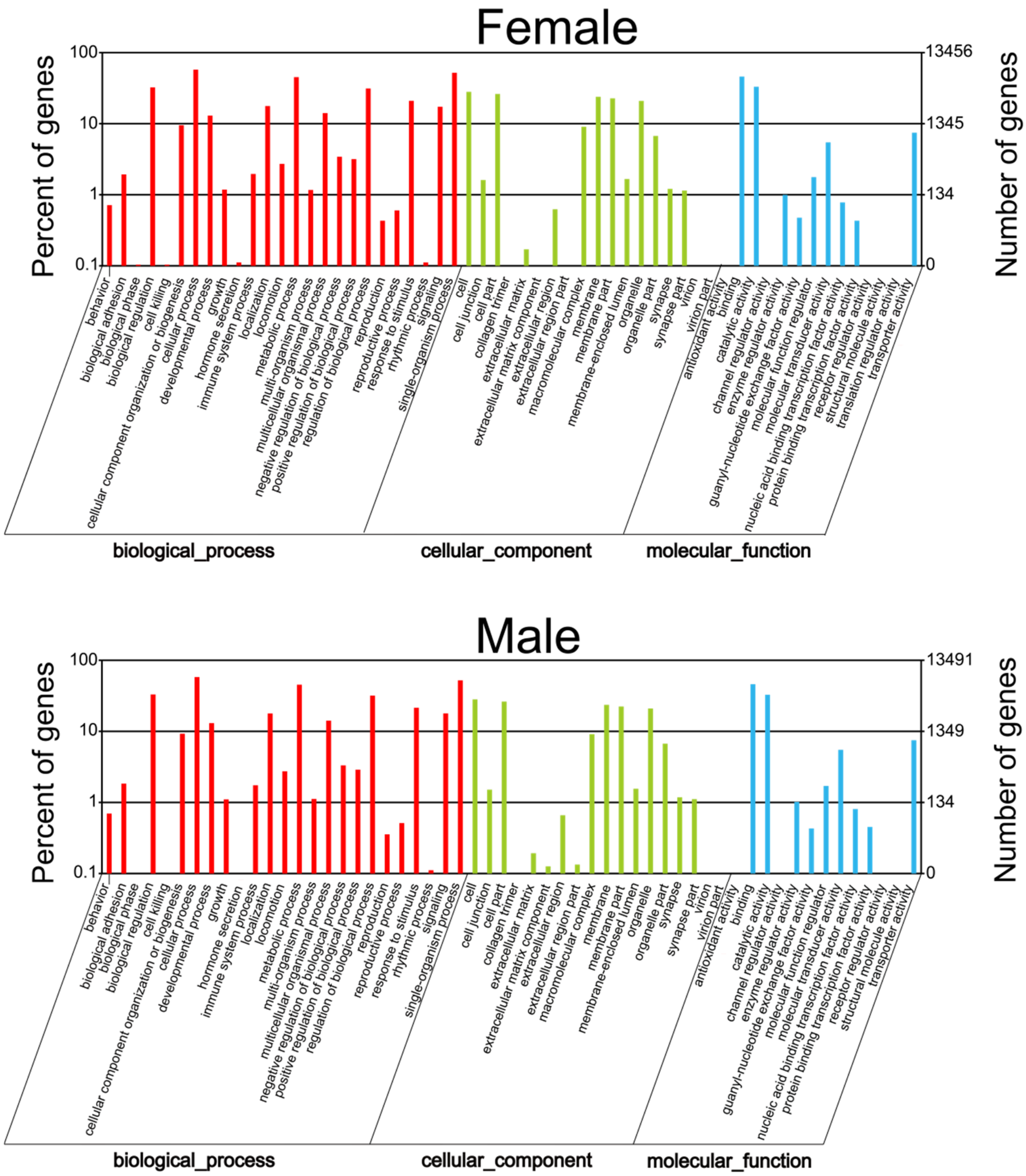

Figure. S3. Gene Ontology classification of predicted genes in *S. argus*. Genes were assigned to three categories: biological process, cellular component and molecular function.

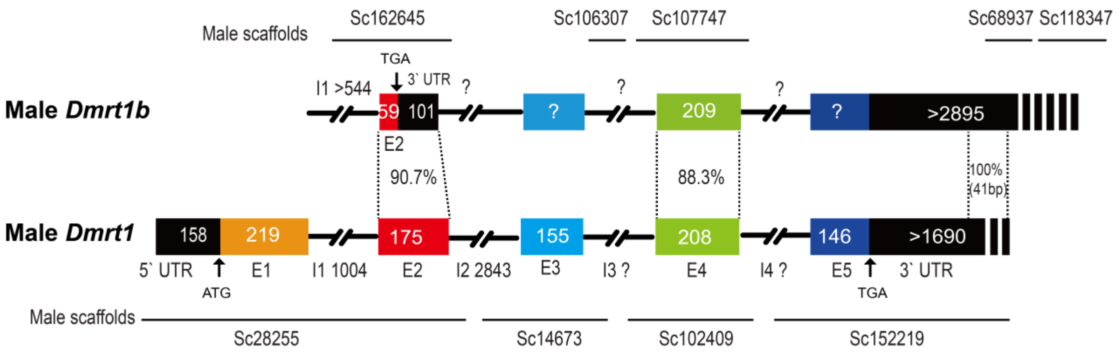

Figure. S4. The *Dmrt1b* gene from male genome. Numbers indicate base pairs (loci) of exon and intron sequences. Percentages indicate the similarity of *Dmrt1* and *Dmrt1b*. Arrows indicate the start and stop codons. Different colored rectangles represent different exons.

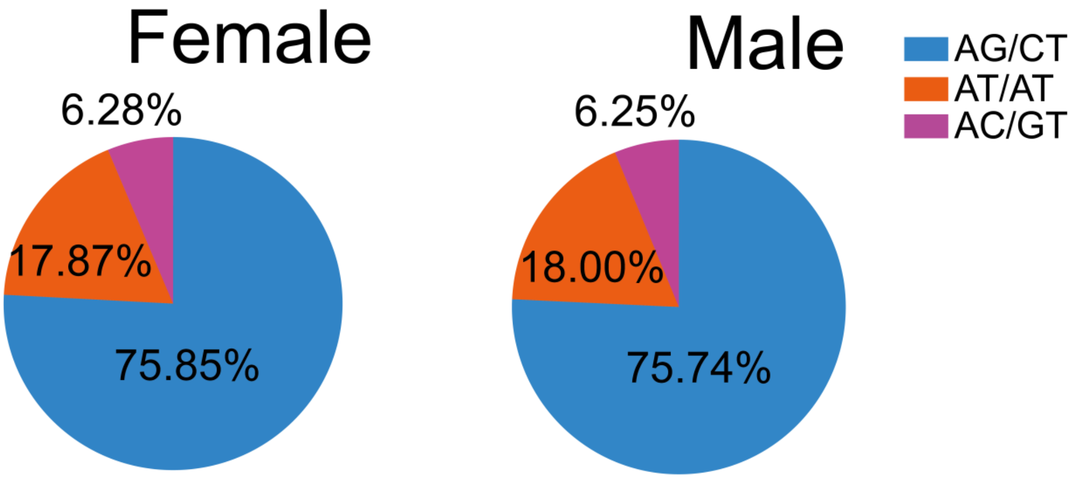

Figure. S5. Percentage of different motifs in di-nucleotide repeats of female and male *S. argus* genome.

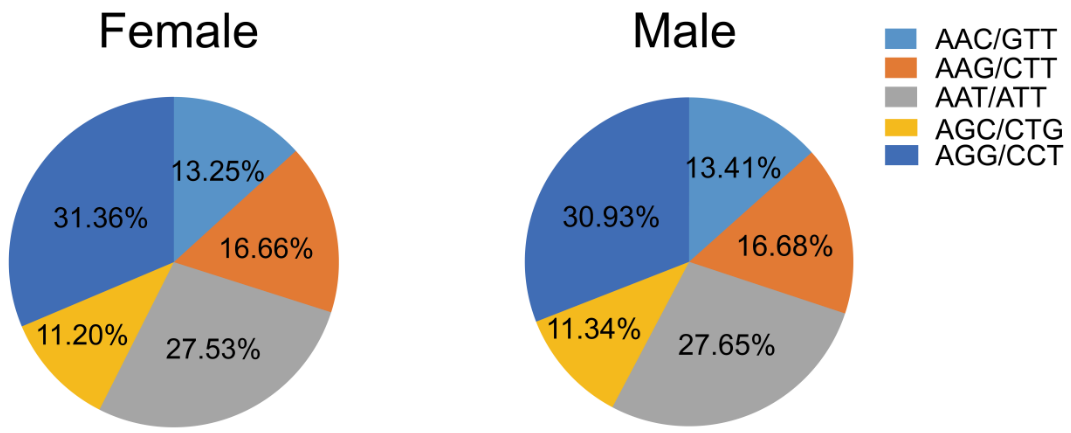

Figure. S6. Percentage of different motifs in trinucleotide repeats of female and male *S. argus* genome.

Supplementary Tables

Table S1. Statistics of top 4 similar species blasted against the NCBI nonredundant nucleotide database.

Species	Percentage (female/male)
Dicentrarchus labrax	0.91 / 0.91
Scatophagus argus	0.49/0.19
Haplochromis burtoni	0.36 / 0.37
Oreochromis niloticus	0.26 / 0.30

Note: Percentage of the similarity more than 90% reads in 5000 reads of female and male *S. argus*.

Table S2. Statistics of KOG function classification of predicted genes in *S. argus*

KOG code	KOG code description	Gene number (female/male)
A	RNA processing and modification	1178/1193
B	Chromatin structure and dynamics	528/543
C	Energy production and conversion	846/877
D	Cell cycle control, cell division, chromosome partitioning	1003/1037
E	Amino acid transport and metabolism	1035/1011
F	Nucleotide transport and metabolism	489/493
G	Carbohydrate transport and metabolism	884/889
H	Coenzyme transport and metabolism	219/208
I	Lipid transport and metabolism	1201/1242
J	Translation, ribosomal structure and biogenesis	725/744
K	Transcription	2901/2904
L	Replication, recombination and repair	659/665
M	Cell wall/membrane/envelope biogenesis	232/239
N	Cell motility	201/181
O	Posttranslational modification, protein turnover, chaperones	3315/3262
P	Inorganic ion transport and metabolism	2092/2110
Q	Secondary metabolites biosynthesis, transport and catabolism	436/438
R	General function prediction only	6357/6386
S	Function unknown	2045/2094
T	Signal transduction mechanisms	11595/11733
U	Intracellular trafficking, secretion, and vesicular transport	2418/2412
V	Defense mechanisms	535/521
W	Extracellular structures	880/877
X	Mobilome: prophages, transposons	0/0
Y	Nuclear structure	134/140
Z	Cytoskeleton	2507/2474

Table S3. Assessment of *S. argus* genome assembly and completeness using BUSCO (Benchmarking Universal Single-Copy Orthologs).

	Genome BUSCO **(female / male)**	**Percentage (female / male)**
Complete BUSCOs	3,055/3,108	66.6%/67.8%
Complete Single-Copy BUSCOs	2,990/3,042	65.2%/66.4%
Complete Duplicated BUSCOs	65/66	1.4%/1.4%
Fragmented BUSCOs	881/838	19.2%/18.3%
Missing BUSCOs	648/638	14.2%/13.9%

Supplementary Sequences

>Male scaffold28255 (Exon1 and 2 of *Dmrt1*)
tcagttcagtcaaacaaaccaaattattattattattgttgttgttgttgttgccaaatattgtcaggaatgcacctcagcagctacacacacagg
ttttcatgttttcagttaatgaaaggatacagttaaacatttgtaaatgggaggtatgtaaatagcgcttgagataacctgacctgctcaccatacct
gactcaggtatttctcaaaagtagatacattgctgtgtgcagatgccaggattgatctcaaatcttgagttaaacggactttctattttaactaaatat
gtctgcaaggttatacagcagattgagaatccaaatcaaggttatacctttctttcagctaggttactactcctgaatttgaagctttttcaaaagatt
acaatctctctatgagcatagagagattgtaatctttttatttatttgctaacatttatgactttgcagattgggattaataatgcaaaatcaatcagcat
acaaattataatgtaatattagatttttcccggggggggggggttaatgagctatggagcagataaaccatatgaagaagttcaaattacctccat
tttcaccagctgcaatgtgaaagtgattaatgcagaaataattgtaatacagtgatatgtataattttcagtattaaagtatattttgctgctatattactt
ttgtccttaggccaaattaaaaaatcgaatatttgaatttgaaaattgtagagctgagctacttgagtcgtagaactatacaagcagaggttcacat
ggaacaatatgcttttggtcattctattttactaaaaaactgtaaacccaattgggtgcagcagactcatgccactactgtgccaactctctccccc
gcttccagcctgtttcctcccaCAAGTCCCGGCAGCAGGGCGGGGAAGGACGGTGACCTTATAGCT
TCCAGCCTGGCACCAGCAAAGTCTTTAACCAGCCGAGCCTGCTGAGACAAACTCCGA
CATAGTATCTTAAGTGACTGTTGTTGTCGTCTTTTTAGTGCTTTATTTCCAGTAGCCATG
AACAAGGACACGCAGAGAAAGCAGGTGCCGGAGTCCACCGGAACTCTGTCCCCATCC
AAACACCAGAAACCCTCCAGGATGCCCAAGTGCTCCCGCTGTAGGAACCACGGCTATA
TATCTCCTCTGAAGGGACACAAACACTTCTGCAACTGGAGGGACTGCCAGTGTCCCAA
ATGTAAACTGATAGCTGAAAGACAGAAAGTCATGGCGGCCCAGgtaacccttattttaccggttagca
cggctagtttgagctgaactgaaaacagtgcttttaaacaaaaagtttgatgtgaaaataaaccgtggtgtgaaacagggaatacaaataaatat
tttaaattatgtttgtatatttttaaatacagtggacaagtttacttgagttgattttcttcattttcatttgtgtatcaacacgtcaaagcttgttccacagc
tagcgtacagagatttactaactttttacagcctgtaggcccactgtgtattactacacttgttactaattgaaattagttaataggtaggcctcggta
tatagagcaatttttccatctttgcgtgttatttatttagtaacattgtacctcgtagtttaaaataacatttttctgtgttttttttaaatgcatccattatcatt
gatcgttagcttttgacagtaagtaacactgggagatagcaggggactaccctgctggccaaggttcaagctgcagatacgcacggctctctg
gccagttcaagaaataataagcaatccaaaaatgcttccttatttacctgagaatttttgagaaaagtttgcaataatgtgtatgtgtgtgtttgcgcc
tcgtaatttttgtgctgttaaagcatatttcgacatgctcctccctgctgtatgtgacagccttttggacatattaaacttaatattcatatagcttatgtt
atcttacaaaatagaatgctataatttcagaaggtggtatcagtaaatgtattctaacaaattaattttcgtttgaagtgttgtactggtagagacaac
aataacatgacatgaatatggcatcagtcctgtccattttatgctctgatttgactaggacaaggactagacagaacactgattgttattaatattatt
attgttatgtatattttctatactttgattgtcttgtctattctgccagaaaatgtcttgtttttcctttcctccccaccagGTCGCACTGAGA
AGGCAGCAAGATCAGGAGGAAGAGCTTGGTATTTGTAGTCCTGTCATTTTGCCAGGGC
CTGAGGTGATGGTGAAGGATGAAGCCGGAGCAGACTGCATGCTCTCTGCTGAGGGAC
GATCCCCGACACCTACCAGTACCTCCAATTTTTCTCTTGCTGCCACAGgtgaggtattgtgagtctt
tagttacacacttgagcccacttgcatgtgtcaaaaccttctcatgttttgtactactaatgtagccaacagctgatcatacagctccacaggtaag
tcaaaaatcttaaaaaaactagagtgggcatgcacatgagtgtaaatatggaacatggaacagtcctaatagccaaagcacattcagatgaaa
aaacatggaaaatgcctcttaaatggtgtaaagctgttttgaagttcaaggagacatttttaggcttcacaaaaatttattgttaaaggttaaaattaa
ggatcacttctgcatcataatgctgcatgctaacactactagctaatgaggcgatagaaattgcctgtcatctgtctctgctgttttctccttgtgctt
catgtgcaaatggttaatcatcagacaagttggtctgtgacatccaattttaacatttcatagctttcactgaaatacttccaaacatcactcttatac
ctactggtcactattctgcctgtgattgaatggtgtctggctgaaattgctgaacatcattagactgatctgtaatttttgctgtgcagatgttcatgg
atagcagctccatctctgtgcccagtccaaaaaaaagtgaacattgactatagaacatggtgaattcacttttatatttattatgctaaatgcagatgt
cctgtttagtgctgcacctaactattatcgattaatctgccaattatttccacaattaatcttttagtctataaaatgttgaaaatgctcacagttcccag
agcccaaagttacatgtacaaagtgcttcttttatccagctgacagacctaagcccaaagaccctttactgtcataaattaaagggaaaagcagc
aaatcattacttttaagaagctggaaccagcagatgttttgcttaaaaaataactgaaacgattaatctattatcaaaatagttcacagctttttttttatt
tgttttttttttttttcaactgaccaattgattaattgaatactgaatactcatgcccatcccttatatctacaacttatttaaacaattttggacattgggc

aagattcattaaactgtgaatgcccaagctcactatattccttgttagtgtgatatcttatgaaattatcaaattctgcatgatgtgtagaacatttgtct
acatgtaaaacatccctctagaatagatgcatctgctttgaatgtttttcttcaatttgtttttgatattcaaaaatgccattaaaaactgcatctgaga
aaaggacttccagtgtttatggactgtggcttttaagacttgtttgctctgttaattattcctttttgtggaacagactgttttgatctttttgaaaaaact
gatcaatagctggctgaagacatgcttccagataccacttatctttaaaactttcttccgtaatttcactttgttttgtgtatttttctgagccacaagtg
ctccaaagtctataaaatggatttactgataaacttgttttaagaacaatggaataacaacttggggaaggaaattaagaagatcgtttttatcagct
ggctttctagagatagtggtggcaatccagttatgtaactttaacaattttacttttagaaaactagaaaacagacctgtgactgcatgctaaaatta
caacaacaaacaaaaaaacaacaacaacactacaatttgtaccgattagcatattttattgttctgttatttagtaatgcaaagcaattaaataaatc
cacaggacatgagtactttcatagggactgtaaataccatagataagacacattttcatgtgaaaagaaatgaaaattcaagttcatttgttggca
ggagcatgaaagcgcatccttgatatctttct

>Male scaffold14673 (Exon3 of *Dmrt1*)
tgtcttcattgtgtaagacagaaaggtcaggttctgcctgaatgtggaatgtagcaagaatgtggccttgttgataaggaaagaacatcggataa
tgagtaatgatgatttgattgctacgttggtcctgacagagcaacccacatttctgtattgtgatgttcatgtgttaggtctgccaagttgacatcag
aaacacctgtcacctgcagatggtgctattgatagcaatttgacctagtggtggaatgaatagaagaatcttatgaagttcttatgttttcttttcctg
tcaactgttcgatcaaaagatctgaggcaagcctagatctatacaatttgaatacatcgaattattttcattcagaggctcattttggaaaataaaaa
caaaatgtctgcttgagctctgctcttgggtgcataacatgacaacatggttaagttttaaaaattagcttattaaccctaattaatccaagcaaaac
ataaactaactaaaacaaatactttaacgcagtgctcagattcatatttcaaatgtgatgatgatctagaaatactgaaatacaaccacaaacatct
cactttagtctctgaaacaatcttgtttgttgtaccttctgtttatgtgtttgtctagGGAGTTGCTCGATGTCCTCCAGCCTG
TCGACTGGTGCCCGGGCTCATACTGAGGAACCATCTGACCTGCTGCTGGAAAACTCCT
ATTACAACTTCCACCGGCCTTTGTGCTACCCCACTTACTATGGCAACCTCTACAACTATC
AGCAATACCAGgtgactcacaacaaccactctgctgtttatcaatccaacagtcaaactctcatttgaagccttttgaggactcatagt
agatatttatacactgtagtgtgtgaaaatccttatttgattaacagcaggttcatgttgatggatgatgggtgatgaataaacccaggaaaaagat
attcatcacatcacattcatattgcttcatttttccaaccagaaacactcttcatttacaaagaaacacaacaaatccttacatttagtgaactggaca
catatgaaatgatggtctgttgtttgtgtgcatgtaagaaatgataccacatttattatttattattatagtattaattttctttcaacagtatttgtcaagg
atcatagacaaaacaatgattttaacttagtcacagtcattaacttaagctaatagcagtttacatagcacaaattagcctagctgcaagcagactg
tactaaacatgttatactttttcttcactgatgcatgacttaaagccaccagtttaagtggcaggtaaggtaagttttaatctgcatgcacatctgcatt
aatctgcatgcacattttctcagtctaacactgttgaaacagtgttgttaatatgcaagtgctacaagttagcagactgctgagtgagtttgttgtatc
tcccgatcaatcaaaatccaaactatttctacacagcttatttattcctgagagggtggtaaatgtcctcattgtctttttcttggctacttaccatcaa
cctacctggtgctaccactgtttgaatgtttaggcaggaggtgagcttggactgaaatggtgatgtggacaagccatgggaatttcaaaatgaa
gatgcatcttaaagataatgattgatcaatgaatctatatattgacctaggctgatggattgttacacctctggtgttggtgtaagtgtgtgtgtgtgt
gtgtgtgtgtgtgtgtgtgtgtgtgtgtgtgtgtgtgttggagtgcagacttgacccacgtcagtctttgagctcaactttgtgaatgcacagctt
gaagggaagaacatggtaccaagtgttactttccacaatattctgtttcaaatgatctttacttaacggaacaaattagttcagatgcctttaagtttg
tgaatttgagtattcattcactttagtgaagctagatgagcagtcttgtcacctaaagagtgtcatttcatgaacctggatttatcattgaatggactg
tccatgaacatgtccatacagtacaggattgttgtatctggtagctgtgtagtatttatgtttatcagactgttatgatgctgtagtgcagtagtaaaa
gtagtctgttctgatttgtttacaggtgtgtgtttagatggtcaggccaacagaaacatccaaatgcttcttgtttatgctacataccagcagtggat
aatgtacataactccattacttgatacttctgatcaatattacaaaatatatgtatataaaatattattaaaatatagaaatagagaaaagtgacaaca
taaatcagtacatttgtgaagctggcaccaacaaaaattttgaatttttgcttgataaacaacagactgattatcaaaactgactgattttctgttaaat
aaatcagttgactaattgtttcagcagcaatgaaagccacgtgcgtgtagaagataatgggcagtaagcaacaataaaaagacaaaaccattgt
gatgaaaagtttatgatctaccaggaccacaacagtggaagaaaaaaaaagacaggaagcatcaccttgatatgacagtgatataacacaaa
gcagttaagaagaagaggttaattttctttgaatctggtagtgatttatacatgcttttatttctttgttttcctaagagatgaatgagagtgtctgcttta
atgttggcacttacactataacataggacacatcggggagacagtacccagtgtcttaccaacacacaaacacacactatacatctgctgtcctc
agctacatccacaagtaacaggacagtaacaacctaaccaaaacagaaaccctggctgaatgaggaggtctctgctgaaagccagggatgc
tgcgtttaggtcaggtgacagactggcactcagggtggcgagaggagagctgactgcaggannnnnnnnnnnnnnnnnnnnnn
nnnnnnnnnnnnnnnnnnnnnnnnnnnnnnnnnnnnnnnnnnnnnnnnnnnnnnnnnnnnnnnnnnn

nnnnnnnnnnnnnnnnnnnnnnnnnnnnnnnnnnnnnnnnnnnnnnnnnnnnnnnnnnnnnnnnnnnnnnnnnnnnnnaagagggccaagacc
gcatatgcccagaggatccagggacaatttacatccaacgatccatggagcatgtggaggggcataaagtgc

>Male scaffold102409 (Exon4 of *Dmrt1*)
ctcagtgccatgaaaatgcatttagaaacaggctctggagatgtctttttatcaagttatcatcctgtcatctatgatttgtgcagaacacctctttg
agttcatgtcagtgaagaaaccaaccctaactgtaaggaaaataataaattacattacacacaattaatcaccaactacactgataccaagtgtgt
aacagtacacaaaatgtgtagttcaacatggttatgcggtcactgttgattttgatgattttggtacagtggtgaaaacacaagattgaattttctttc
catttgttttaaaaaccacctaacagtggctcagtcgttcattgtcataatcataaaaaatagtcatagtcataaaaattaaggcactccatcaactg
tccactatcaagaagtagtctaaaagaaaacaaaagggaatatatcaacaaagctatatgcacataaaccagcactgacaggatattatgtttaa
atcttatggactctgggtctcactgagtattgtagcatatctgaagtgatgaacactcccagggaacttacttattcatttggcctttggctgcttgca
cacagaagaaagactgacatgcacccgaggctgttaaaacaacctctagtaatagacacactagagctatttctgcaactgatgttgcctggct
ttactccatgtccactatagcttagtgcctctactctgagcccatccagcctgcagaggaaactcatttatacaacccgtatctgtgaagtcattctt
tcggtcgctacccaaagctcctcaccatagactttccagctccgctcaagtattactgttgatgctgcaccaatctccctgtcgaacatctcagaa
caacgtctattttgtgcattatttggcatactgatttggcatactgaacatacagacacggctttcaacccaccagtaacattgagaaaggtttatga
actgttaatatggcttcacattgacatttgggattactcagtgtggatccttctctgtgatcaaggcaaaacctgccttgctcacagagaagagca
actttcaataagacagttttgtttggtgttgtatgtgtcctgaaaactgtaagcttagtgcactctcaagatcccagttcaatctctaatgtatttttcac
agcctaatacaagagctgagtgtagcttaaataactctttataaagctgtcagagaattgatggtgtggacaatacaggtgacctattacaggct
gacagaaatcaaattccataaaagtgaaattgaaaaccctgtcagacggcctcgctgctgagtagacacctcgttttaaagttttcagaggactg
aagaaagccagcacagcgcgttgtagctcatttttacaaaagggcacaactcatgaattcaaacattgctaattctctattattattttacctctatat
ttcgtcctttcggattaggttgtttaagtattgcctgtgaatatctcatcaccagtttagaggactatacagtcaaacatgggtgaattgagtcatagt
acagtggtattcatattgtatttgaaatttcaaatatagaaaaggaaccttctttaaaaaaagttatatgattaaaaaaacaatcatacaatgcatgac
ttggatgttgtctgtgaaatacagcacatgaagacaattctttatatcagttccaattcctgtccttgtgttttcttggatgaagCAAATGCCC
CATGATGATGGCCTCCTGTCGAGCCACAACATGTCCTCTCAGTACTACATGCATTCCTAC
TACCCAGCAGCCACCTACTTGACTCAGAGCCTGGTTTCCACCCCCTGTGTGCCAGGTG
TCTTCAACCTGGAGGACAACAACAGCAACAAAAACAACAATAACAACAATGACAACA
ACTGCTCTGAGGCCATGGCTGCATgtgagtgacacacaaaaacactcacacacacttctcaggccccataaagtgttact
caggctttatccacagtgtagttaagtgcgttcaagggagaattcaactcaagtcaggagcgccataaaatttgcaagatgaaatttgtccaaag
tgaagcttttatactctttactatgatttctttttcctttttaatgaagttcaaattcaaattcaaattaaattcatgtcttgcaccaatttccaaatgagaat
ttccatgaatgaatcaagaggagagtgtcagagcgtgaggattgaagacagtgaagccagccttaaaatgaataaaacctaacctaaaccatt
gttccatcttttgttttgattgagagacccctagcagccaaaaatatgtgctttgtatttaaaggatgacagaaagttaaataaaattatgctcagcta
attggcaaaataccccatacctgttggctttaatttgcagccatcatacagaaaggaaaacattttcaaaacaaccactaaactggcaaaga

>Male scaffold152219 (Exon5 of *Dmrt1*)
gaggtttatttttcaagtgcataaacatactttaccagagcacgacaacaatttaatgtttattgttatgtgttttaataatttttaaccataataatattg
ttatacaaagccttttattttgtgacatactatttattattatgtgcaatgttgaaatttgataaatattaaaaaaacaatttaaaaaattagtcatgtcaca
gttatcctaccaaaataaaagcatttatcctcacacagtaaatactgtaaaattacatacaggttgcctgtgtagtgaagaggctcttgactgaaaa
gaggaaaaaaactagttggtccttgcttactatcacacctccgcatatctgtccaataattaggtgaagtagccataactgttgctgtgtgactata
gtagccagctttgacaactttatgaagtagcctccaaatggactttgactggcaaaaacacagaattgagcagtttcatgttaaaattcagtgtttc
ttcaacactcttcagtggacacaggatggcagcgagttgagctgatactttggtgggcttgtttctctgacaacttaagatatgacgaagatc
tttgatccagttcaagacaaagttaaaaacaagcaagatctaagaatcttatagacagtaggtggctgaaaatgttgtctggccatcatgccagc
attggacacttagggaaaacgctgctgtttcattgtctactttgaaaagttaaaaataaaccggattctgtgtgtctcatttctgcagCCTTCT
CCCCCAGTCACATCTCCATTGTTCACGACTCCGCCTGCAGGACCCCGCTTAACTCTGAG
GTCAATGTTGAATGTGAGGCCAGCGATGAGATGCCAAACTTCACTGTCAGCTCCATCAT
CGATGGTGACGCTGCCAAATGAAAAAAGCTAAGGATAAAATTATACAACAAAGAGCAG

TTACTTTATGTTCTTGAAAAGCAGTTTCACAAGTTTATTGTCACTATAGGTTTTTCTGGT
CTTTAGGTCTTTCTGTCCAGGAGGCATAGTGATGTCTGTTGCATTTAGTTAAGAGTAAA
GAATGTAGGTTGTTAAATCTTCTGTTGAAAACAGTTTGTCAGAATAGAAAATGCATTGA
AATTAAAAAAAAAAAAAAAGCAAACAACAAAAAACTGTACAAGATTCAAGAGTCTTT
ATTGTCATTATGCAAGCATAACGAAATTTTGTGCAGATTCTCAGCTTAAAAACACACTA
ATAAATAGTCAGAGAAAATTAAAATTACACACTTAAGAAAAAAGAGTTCACAAATACC
AAAATGCAAATATTTGAGAAGCCTCTCCTCAAAATTAAGCTTTTGATGCTTTGTACAAA
CAATGTTAAAGATTTTCAAACATGCACTAAAAGTGTCTCTTTATTGCCACTTATCAAGA
GTTCATCAAGTTAGTAAAACCAGATTGTAACTTGTCGTGTATTTGTAAAGTTGTACACTT
TTTTTTTCCTCTTCAGAGCACAATTGTTAGGCAAAGTGAACTTCTTTTTGAATGCCCAG
AAGATAAAATAACCCAAAATACAAAATAATGATATTATAACCCTTTCTGGCTTACAACAA
ATAGGACTAATGTAGTTTTCAAACAGTATCAGGGATGTGCTTCTGTTAACAGTGAATGG
GCAGTGAATGATTATTTAGATACAGTGAACTTTCTTTTAATCAGAATATATACAGAAATC
ACTGCTGCTGTACATTTCAAGCACTTGGCAAAAGTTTTTCTTTAATACAGTCATTTAAAT
GTAGAGTGACATGCAATTCTTTGTGTAGTATTCATATTTGTTTTTAGTATTAGTTTGTCAG
CCTTTTCAAATCTGTTTTGGTCCAGTGAACTAATAAACAAAGTTGATCCCAACCTGGCC
TAGCAGGTTGGGATCACTGGTAGAGGTTAACAAATCAGAGAATGATGATCTGCATATAA
TTTCAACACTCGATTTTAAAGTATACTTAGGACCATAGACTGTATAATAATTGTGGACTA
CCATTTGAAGCCTCGAGTGTGGCATTGTGGATCTGATTGAGAACCTGAGGACATGCCAT
GGTAGTCTCTTGTCAATCGGAAGTTGGCACACCTTAAAGCATTCCCAGCTTCATTGTCT
GGACCATAATTTACTCAATGAACATCATGCTGTATTGGTGAAAGTAGCGACTCCTTAGG
GTACTGTTCTCTTAAAATTATATGTAGTCAGACTTGTTTCTGAATCCCCTGCTGGACATT
AGAAAGAAAAAGTTTTAAAGCACTGTGTATCAACAATAACAATAACTTAGCAATAAAG
TCTTGTCTCCGTGGCTTCACTTTTCAGACACAGGGGCACAGTCCATTTATTATACTGTCT
ATGGTCAGCACCTGCTTCTCTTTGAAATATTGTCATTAAAATGCTGTTGAAACAACGGT
TAGGAGCAACTGTCTTGTCAACAATACTGTAACCCAACAGATGCTGTTACTGATATACT
GATATTTCAGAGTACAGTACAGTATAGAGTGTAAAATTCTGATAATTGTTCATACATACT
TTTTTTCTTACAATTTGATAGGGAAAACATTTTAAATAATTGTAACCATTGTAC

>Predicated amino acid sequence of *Dmrt1*
MNKDTQRKQVPESTGTLSPSKHQKPSRMPKCSRCRNHGYISPLKGHKHFCNWRDCQCPK
CKLIAERQKVMAAQVALRRQQDQEEELGICSPVILPGPEVMVKDEAGADCMLSAEGRSPT
PTSTSNFSLAATGSCSMSSSLSTGARAHTEEPSDLLLENSYYNFHRPLCYPTYYGNLYNYQ
QYQQMPHDDGLLSSHNMSSQYYMHSYYPAATYLTQSLVSTPCVPGVFNLEDNNSNKNNN
NNNDNNCSEAMAASFSPSHISIVHDSACRTPLNSEVNVECEASDEMPNFTVSSIIDGDAAK.

>Female scaffold83349 (Exon1 and 2 of *Dmrt1b*)
atatatatactgtcaataaagctgattcttcttcttctttactccactttatttatttgctaacttttgtcactttgcagaatgggattaataatgccaaatca
atcaacatacaaattattattattattatgtttagtattaatgggtaatgtttattatatttattattatcataatatatagtatccattttcaccaggtgcaac
gtgaaagtgataaatgcagaaataattgtaatacagtgatatgtattattctcagtactaagtagtaaatatattttgctgctatgttatttttgcagtta
ggccaaattaaaaaatcgaaagtttgaatttgaaaactgtagagctgagctacttgagtcgttgaactatacaagcagAGGGACAATC
CGACATAGTATCTTAAGTGACTGCTATTTCCAGTCGCCATGAACAAGGACACGCAGAG
CAAGCAGGTGCCGGAGTCCACCGGAACTCTCTCCCCATCCACGGGCCAGAAACACCG
CAGGATCTTCAAGTGCTCCCTCTGTAGGAACCACGGCTGTATAATTCCTGTGAAGGGGC

ACAAACGCTTCTGCAAGTGGAGGGACTGCCAGTGTCCCAAATGTAAACTGACAGCTG
ACTTGACTGACATTCAAGgtaaccgttattttaccggttagcacggctagcttgagctgaactgaaaacagtgcttataaacaa
aaaaaaagtttgatgtgaaaataaaccgtggtgtgaaacagggaatacaacatggaaaggttcacataactattttctaattatgtttgtatattttta
aatacagcggacaagtttactgtagttgcttttcttaatttctatttgtgtatcaacacgtcaaagctagttccacagctagcgtacagagctttacta
actctttacagcctgtttaatgagtgggcccactgtgtattactacacttgttactaatagaaattagttaatacagtaggtaggtctcagtattaaag
agcaattactttttccatcttttcatgtcatttactttgtaacattgtacctcgtagcttgaagtaacattttagtgttttttaaacgcatccattatcattga
tcgttagcttttgacagtaagtaacaccgggaggtagcaggggactaccctgctggccaaggttcaagctgcagatacgcacggctctctggc
ccgttcagaaaatcataagcattccaaaaatgcttccttatttacctgagaatttaacgcatgagtaatttttgagaaaaatttgcaatactgtgtgtg
tgcgtgtgcgcctcgtaattgttgtgctgttcaaagcatatttcgacatgctcctgcctgctgtatgcgacagcctttgggacatattaaacttactat
tcatatagcttatgttatattacaagatagattgctattatttgagaaggtggtatcagtaaatgtattctaacaaattcaatttcatttgaagtgttgtac
tggtagagacagcaataacatgacatgaatatggcatcagtcctgtccattttatgctctgatttgactgggacaaggactagacagaacactga
ttgttattattgttattaatattatcattgttatgtatattttctatacttctgattatcttgtctattctgccagataatgtcttgtttttcctttcctccccacca
gAGGGCAAGAAAAAGGAAGCAAGCTCGGCAGGAAAAGCTCGGTATTTTGCCAGGGCC
TGAGGTGATGGTGAAGGATGAAGCCGGAGCAGACTGCATGCTCTCTGCTGAGGGACG
ATCCCCGACACCTACCAGTACCTCCAATTCTTCTCTTGCTGCCACAGgtgaggtattgcaagtcttta
gttccacagttgagctcacttccatgtgtcaaaaccttctcatgtgttgtactactaatgtagccaacagctgattgtacagctccactggtgagtc
aaaaaccttaataaataaactagagtgggcatgcacatgagtgcaaacatggaacatggaacagtcttaatagccaaagcacattcagatgaa
aaaacatgcaatgaaaacatgaaaagaaaacttcagacctgcgactgcatgccataattacaaaaacaaacaaacaaaaaaaaaaaccaaca
acacttcaatttgtaccaactagcatattttattgttctgttatttagtgatgtaaagaaa

>Predicated amino acid sequence of *Dmrt1b*
MNKDTQSKQVPESTGTLSPSTGQKHRRIFKCSLCRNHGCIIPVKGHKRFCKWRDCQCPKC
KLTADLTDIQEGKKKEASSAGKARYFARA.

> Female scaffold545689 （Contains homologous sequences to the exon 3 and 4 of *Dmrt1*）
tagcatattttattgttctgttatttagtgatgtaaagaaattaaataaatccacaggacatgagtactttcatagggactgtaaataccatagataag
acacattttcatgtgaaaagaaatgaaaattaaagttaacttgttggcaggagcatgaaagcgcatccttgatatctttcttgggagtggtgtgga
ggcagacacagataaccatgttccctgtcagcctgatagcaaattttagtacttggaatgtgctggtcaaggctgctcacgtcttgctgtgattct
gctttgtgtctctgtgagaatgaattttgggtctctggtttggcgacagagtcgaaagactgaggtggaaaaaggagacgagcactctgactca
ctgatgacaagaattagtgcaggaacaacaaatgtcattatttttggcttcagcggtatatggtgtcttcattgtgtaagacagaaaggtcaggttc
tgcctgacaagaatgtggccttgttgataaggaaaaaacattggataatgaataatgatgatttgatggcccacgttggtcctgacagagcaaccc
acatttctgtattgtgatgttcttgtgttaggtctgccaagttgacatcagaaacacacgtcacctgcagatggtgctattgatagcaatttgaccta
gtggtggaatgaatagaagaatcttatgaagttcatggtttcttttcctatcaactgtccgatcaatagatttgaggcaagcctagatctatacaattt
gaatacattcaaaggctcattttggaaaataaaaacaaaacgtctgcttgagctctgctcttgggtgcataattcatgccaaagcataaaccaact
taaactagtacattaacgcagttctcagattcatatttcaaatgtgatgatgatctagaaatactgaaatacagccacaaacatctcactttagtctct
gaaacaatcttatttgttgtgccttctgtttatgtgtttgtccagggagTGCTCGATGTTGTCCTCCGCCCCGGCGACT
GCTGCCCAGGCTCATGCTGAGGGACCATTTTACCTGCTGCTGAAAACCTCCAGTTTCA
ACTTCCACTAGCCTTTGGACTACCCCACTTACTATGTCAGCTTCTGCAACTCACAACAA
CCACTgtgccttttatcaatcaccagtcaaactctcatttgaagccttttttgagggcttatagtagatatttgtacactgtagtgtgtgaaaatcct
gctctgattaacagcaggttcatgtttgcctaaagtatgttgctgaggttgttgatgctcttgctcctatacctaccttatgaaaggttgaggattcag
cctgctgcaagagtcgtatgcaagagttagaaaaatagagcagaccgcagtactgcaactaatgattattttcattatcatcataattaattggag
gattaattaattatttgatgaataaacaaagtaaaaaatattcatcacatcacattcatattgcttcttttgcccaaccagaaacactcttcatttacaa
agaaacacagcaaatccttacatttagtgaactggacacatatgaaatgatggtctgttgtttgtttggatgtttttggtgcagactaccgaaaacat
ccaaacacttcttgtttatgctacatactagcagtggataaagtacttaactccattacttgatacttctgatcaatattccaaaatatatgtatatataa

tattattaaaatatggaaatagagcaaagtgacaacataaatcagtacatttgtgaagctggcaccaacaaaaatttagaatctttgcttgataaac
aacagactgattatcaaaactgactgattttcagttgactaattgtttcagtagcgatgaaagccgcgtgcgtgtagaagataacgggcagtaag
caaaaataaaaagacaggaagcatcactttgatatgacagtgatataacacaaagcagttaagaagaagaggttaattatatttgaatctggtag
tgatttatatatgcttttatttctttgttttcctaagagatgaatgagagtgtctgctttaatgttggcacttacactataacataggacacagacagtac
ccagcgtcttaccaacacacaaacacacacaatttgtccctttgtccctcattcacccctctgcatattctcactgatttatgctcaaatgtatctctat
tgtgtccagtgagatgagcttgtgcatgaaataaattagatctataactctccggtgatctcagatgaggtggtttgtttttttatgaagaacatttgt
ccacccctgctctctcctctccaaccccctcgcgttgctccaatagcaacatctagttttggggtcactgcctcccctaacctgccagcacacag
cagaacctgaattgcaaaggttaatggtattgtctgtcgacagaggaacaaaccgctatgcctcagtgctcgaaggagaaagtgagcacatta
atgctaatcagttagcagtctttagcataatttagccatcctcctttgtgatttatgatattttcagtgaagttgtcaggttgagaggacgatggcccg
ctgttattcaaattcagggcgtcctttttttacacttgcatcaagagctgattatgtgaagatttgttttagtgtcacacagttgcacaatttgtgactag
aaatttattgttatgatcacaaaaagtgatagaatatttctttagggataacaaaaagtatttaatattaccacttctaataataattaccacatctcctc
aggtaaaaataaataaatgaataaagccgtgtctcctctgtctcctttacctgtcctaaacctccacaatttaatgggcaatagtccaattttagcat
cctcaactgaagtgattatccttgggtgactcaattttgattttgtctccatatttgctgtgaagcacttatcgctggggtcgttctgccctattcagag
tgcacttgtctgtcaaacaatgttgggctgtgacgtttttgtatacatatatacttatacacacatatatatttatatatatgtacttatatatactgttaat
caaaacccataatgaaacactgcagtctttatcagtgtgaggccatctacctcatcctctcctgtgcattcagtggcgcttggttgtcatgcaggt
ggatggtgataatataagggtggtgtagagcttccagacaggagatggtgccagataagaagacaggccctttcagggaggtagagaagcc
cccccaggagtcagcaagttagcccatgagctaagaggaagtactgtacctgataccaagtagcatttaccattgctgtactctcagtataactt
attccattatgttgtgcgcacactgtgttcttgctttaatgatatcattggatttactgagttgttgttaatctcatttcatccataacccatagcaaaaac
tgcctgttcacctctgccaaaattgtgcatttctagtttatgcattcacttgtttacttactaatcctcagtgccatgaaaatgcatttagaaacaggct
ctggagaggtctttttatcaagttatcatcctctcatctgtgatttctgcagaacacctctttgagttcatgtcagtgaagaaaccaaccctaactgt
aaggaaaataataaattacattacacacaattaatcagctaatgttacctgactttactccatgtccagtatagcttaatttactggacaaccacaac
aagttcaaaaagaggatagtatgagaaggtcctatagccttgactctgttctggatgtaacctaccacgcccagaaaacctctgaaggagggt
gctgaggagatatccagatcagatctgcgggctgtcttttgaaggagcagtggctctactctgagcccatccagcctgcagagggaactcattt
atgcagcctgtatctgtgaagtcattctttcggtcgctacccaaagctcctcaccacagactttccagctcagcttgagtattactgttgatgctgc
accaatctgcctgtcgaacatctcagaacaatgtctattttgtgcattatttgggatactgaacatacagacacgactttcagcccaccaataacac
tgagaaaggtttatgaactgttaatatggcttcacattgacagttggggttactctgtgtggatccttctctgtggcagaacctgcctttttttgagcaa
ctttcaataagacagttttgtttggtgctgtatgtgtcctgaaaactgtaagcttagtgcactctcaagacccagttcaatctccaatgtatttttcaca
gcctaatacaagagctgagtgtagcttaaataacagtttataaagctgtcggagaattgatggtgtggacaatacgggtgacatattacaggctg
acagaaatcaaattccataaaagtgaaattgaaaaccctctgcgaggcggcctcgctgctgagtagacagctcgttttaaagttttcagaggact
gaaagcagcgtgttgcagctcatttttagaaaaggccacaacccatgaattcaaacactactaattctctgttattattttacctctatatttcatccttt
cagattaggttgtttaagtattgcctgtgaatatctcatggccagtttggagaactgtacagtcaaacatgggtgaactgagtcatagtacaatggt
attcatattgtatttgaaatttcaaatatagaaaaggaaccttctttaaaaaaaagttatatgataaaaaaaaacaatcgtacaatgcatgactcggat
gttgtctgtgaaatacagcacatgACGACAATTCTTTATATCAGCCTCCTGTCGAGCCGCAACATGTC
CTCTCGGCACTGCATGCATTCCTACTACCCAGCAGACACCTACTCGGCTCAGAGACTG
GATTCCACCCCCTGTGTGCCAAGTGTCTTCAACCTGGAGGACAACAACAGCAACAAC
AACAATGACAACAATGACAACAACTGCTCTGAGGCCATGGCTGCATGTGAgttacacacaaaa
cactcacacacacttcccaggccccataaagtgttactcgggctttatccacagtgtagttaagtgcgttcaagggagaattgcactcaagtcag
gagcgctataaaatttgcaaatgaaatttgtccaaagtgaagcttgcttactttttttactttttaatgaaattcacattcaaattaaattcatgtcttgca
gcagtttccaaaagagaatttccatgaatgagtcaagaggagactgtcagagcgtgaggattgaagacagtgaagctaaccttaaaatgaata
aaacctaaaaacaataactcccatgatcccattgttccatcttttgttttgattgagagtcccctagcagccaaaaatatgcggtttgtatttaaagg
atgacagaaagttaaataaaattatgctcagctaattggcaaaatactccacacctattggctttaatttgcagccatcatacaaaaaggaaaaca
ttttcaaaacaaccactaaactggcaaagacctgatggcacagctctgtttctttttgtcaggttcaaggatgaagcagatctgatctctgatttctg
gcactctacaccaccagtctcagattgaaatctcagctgagcatggctgctgacggtggttgcctggatttttttttttt

> Female scaffold49157 (Contains homologous sequences to the 3` UTR of *Dmrt1*)

GAAGGCCAGGCTTATAAAGTGGAGTGAATAGATAGAACTTATGTTGTGATAAAACACCT
GACAGAACTCAGGTGTGCCTCAGCTGCCAACAGGAGGAGTCAGCCACACCCCCTGCC
ACCCACAGGCTCTGCAAAAGGAACAGAAAATGGAAAGGTAAACAGAGAGAGCACAA
AAAGAAAAGCACAACAGAAAACATACTACACCAAACCATAACAACAATAAAGCCTTG
TCTCCATGGCTTCACTTTTCAGACATTTATTATACTGTCTATGGTCAGCACCTGCTTCTCT
TTGAAATATTGTCATTAAAATGCTGCTGAAACAACGGTTAGGAGCAACTGTCTTGTCAA
CAAAACTGTCACCCAACAGATGCTGTTACTGATATATTGATATTTCAGAGTACAATAGTA
TAGAGTGTAAAACTCTGATAATCATTCATACGTACTTTTTTTCTTACAATTTTGATAGGG
AAAACATTTTAAATAATTGTAACCATTGTACTGAGCAGGATAATTCATCAGACCATTTGT
TTCTAAATGAGCTCAAACATGTCTTGTCTTTTCTGTACAATAATTCCATGTCAGTCATCA
GGTGACACATACAAATATATCTGCAATAAGCTATTTTTTCTTGTACAATTTATTGGTGGAT
GACAAGACACCTTTTGACTTGCAACATTGGAAAGTACTCTGAATTAAATACTGTGATTT
ACCTCAAACAACGATCGGATCAATGGGAAAATCAGATGATTGCGAAGCTGCTGAAACT
GCACACGTTCTTTCTTTTTCTGGTATAGATGAAAGTTAATTAAAGCTCAGAGCAGGGAA
CCTGTCTGACAGAGTGTCTCCTCCTGCAGGACAATGTGCAATTCGAGGGTTAGGGGGG
ACAAAAAGCCAAACATGAATCATATTTCCCTTTGTTGTGTTGGTGTGTGATGAGCAACG
ACTGCTTATTTATGGCGCTGATCCGGCAGCACCGTTTATACCACAGAATTTGCATGTCTG
TTGTATATTTAACCCCTTCTGGCCAGGAGTTTTGCTGTGCACACGGGTTACTCTTTACAC
ACTTTGGTACTGCCTCGTAGATACAGCAGAGGATGAGGGGGTGAATCTGTAGCAGTGA
TGTGTAGAGCAGGAATATTTGAGTTTATTATCTGATTAAGTCATCTAGAAAATGAGTCAG
CATCTAATTCGAGTCGTTGGTCAGTCATTTAAGGCATTTTCCAAGACAAAAAAGTAAAA
CATTTTATAGTTTCAGCATCTCTTTGGATTTGCTGTTTTTTCTTGTCCTGAGTTGTAGTAA
ATTGAATATCTTTGAGTTCTGGACTGTTGGGATCTGGCCATTTTTCATGTTTGTTTTGTG
ATGATTTACAGACCCAGTAACATTAATCAAACCCCAAATCAAACTAAAGTTGTTTTTA
CTTCATGAACTAATGATAGTAATCCTGTTTTGCCCACTTATGAGTTGTGTTGTTTAAGTG
TTAATTAAAAAACAACACAGATGAGATTTTGCCAATTTCTCTGCATTGTGAAGCTGACT
AGTACATGGCTGTATTGTATATTGCTTGTGTTTTATGGAGATTCATTTCAAACTGCATGCT
TTGAAAGTGTTTATTTGTGTTTGTGTTAGAATAAGAGAGCAACAAAAGTAATTTGGAGT
CTTGGAGTTTATGTCGCTACAAGTTTTAGGAAAATAAAAACGCCCTAAGTGATGAACTG
TAAATGTGATTGTGTGTGGTGAAACAACAATATAAAACAAAGAAAATTACTTGTATTTT
TTTCTGCTGCGCTGCGGGAACATTCATTGGATTCTGCCCCATCTGTTGTAATCAATAACG
CGACCACCTTGTTTTTTTATGAAACGCACTTACTCTTAATTTCATCCACCAGTGGTTTTA
GAGAGAAGAATGTGGAGTTACCTATAAGACTTTGACATTATAAATCACCCCTTGAGACC
AGATTGTACAGCAGTATCGATTGATCCTGATATATCACAGAAATTTACTGTCACTTCTGT
TTTCAATTAAAGATACAATCAGTCAGATAATGACTTAATTGGATTTTACAGAAGATTGAG
TTTTATTGCCCCGTGCTTTACGAGTTTAGTTAAGGAAGGCCATTTTATCATGGTTGTCAG
GGGTGAGCTGCTGTGCTGCCTTGTCCTTCTGTAGCTGCTGCTGCCCTGTGTACAGCGCA
GGGGAGAAAGTGTCACAGGATTGGTTTTCCTTGAGCTGCCTCCACCTCCATGAATCAT
GCCCTCCCATAATAGATAAAATCTGCTCGGATCCCACCGTGACACCTCCTGGTGGAGTG
ATGGCCGCTCGACACAGACGAGAAGTGTGGAAGAGGACGCCGTAAGATGTTGGTGTAT
ACTTGTGTATACTTTGGATGTAGTTTCATCTGAGTGATGTAGATGAAATATGTGTGATGA
GGTCATCATACAGGATCATACAGGATTTGGGAAGCTCTTTATGCCTGTAGGTAGGGCT

GAACATGTTCAATAAGACAAAAATTGGTCTGAAATGTGTACCACTTGAAGTTTAGGCA
ACATGATCTGCAACTTATTTGCATTCTCTCCTTTTATTCCTCTTTACCTTTTTTTGGTGAA
AATCTGTAACAGCATACTTAATGCTGAGCCTTAGGTTAAAGTTTAGTATATACCTTGTTT
AAATGTTGAAGATTAGTTAGCAAACCAGTACAAACTATATGCTCATTCAATAAAGTACG
TGACAAGGCCTGTTAATGTTGACAGACATCTTCTTTGGCTAAATACAACTGATTGAATG
ATTCAGATATTAATAAATATTATTCTGTTTTCCAAGGTTTGATTATTGTGTGTATTTGTTCC
AAATATTTATATGAAGAAAGATCTGCTTTTCTTTTTTATGACGTGATCTGTTGTGGGTTC
AATACCTCAGCGTTTACTGTGATATATTCAGATTTTTTGAATGAGAGATCAGAAATATCA
GTTTATCGTGATAGATCTACTGGCGGATTTTGGCATGTTT

>Male scaffold162645 (Exon 2 of *Dmrt1b*)

tggccaaggttcaagctgcagatacgcacggctctctggcccgttcagaaaatcataagcattccaaaaatgcttccttatttacctgagaattta
acgcatgagtaatttttgagaaaaatttgcaatactgtgtgtgtgcgtgtgcgcctcgtaattgttgtgctgttcaaagcatatttcgacatgctcctg
cctgctgtatgcgacagcctttgggacatattaaacttactattcatatagcttatgttatattacaagatagattgctattatttgagaaggtggtatc
agtaaatgtattctaacaaattcattttcatttgaagtgttgtactggtagagacagcaataacatgacatgaatatggcatcagtcctgtccattttat
gctctgatttgactgggacaaggactagacagaacactgattgttattattgttattaatattatcattgttatgtatattttctatacttctgattatcttgt
ctattctgccagataatgtcttgtttttcctttcctccccaccagAGGGCAAGAAAAAGGAAGCAAGCTCGGCAGG
AAAAGCTCGGTATTTTGCCAGGGCCTGAGGTGATGGTGAAGGATGAAGCCGGAGCAG
ACTGCATGCTCTCTGCTGAGGGACGATCCCCGACACCTACCAGTACCTCCAATTCTTCT
CTTGCTGCCACAGgtgaggtattgcaagtctttagttccacagttgagctcacttccatgtgtcaaaaccttctcatgtgttgtactact
aatgtagccaacagctgattgtacagctccactggtgagtcaaaaaccttaataaataaactagagtgggcatgcacatgagtgtaaacatgga
acatggaacagtcttaatagccaaagcacattcagatgaaaaaacatgcaatgaaaacatgaaaagaaaacttcagacctgcgactgcatgcc
ataattacaaaaacaaacaaacaaaaaaaacccaacaacacttcaatttgtaccgattagcatattttattgttctgttattta

>Male scaffold106307 (Contains homologous sequences to the intron 3 of *Dmrt1*)

tctgctttaatgttggcacttacactataacataggacacagacagtacccagcgtcttaccaacacacaaacacacacaatttgtccctttgtcc
ctcattcacccctctgcatattctcannnnnnnnnnnnnnnnnnnnnnnnnnnnnnnnnnnnnnnnnnnnnnnnnnnnnnnnnnnnnnnnnnnnnn
nnnnnnnnnnnnnnnnnnnnnnnnnnnnnnnnnnnnnnnnnnnnnnnnnnnnnnnnnnnnnnnnnnnnnnnnnnnnnnnnnnnnnnnnnnnnnnnnn
nnnnnnnntaaattagatctataactctccggtgatctcagatgaggtggtttgtttttttatgaagaacatttgtccacccctgctctctcctcnn
nnnnnnnnnnnnnnnnnnnnnnnnnnnnnnnnnnnnnnnnnnnnnnnnnnnnnnnnnnnnnnnnnnnnnnnnnnnnnnnnnnnnnnnnnnnnnnnn
nnnnnnnnnnngcgttgctccaatagcaacatctagttttggggtcactgcctcccctaacctgccagcacacagcagaacctgaattgcaa
aggttaatggtattgtctgtcgacagaggaacaaaccgctatgcctcagtgctcgaaggagaaagtgagcacattaatgctaatcagttagcag
tctttagcataatttagccatcctcctttgtgatttatgatattttcagtgaagttgtcaggttgagaggacgatggcctgctgttattcaaattcaggg
agtcctttttttacacttgcatcaagagctgattatgtgaagatttgttttagtgtcacacagttgcacaatttgtgactagaaatttattgttatgatcac
aaaaagtgatagaatatttctttagggataacaaaaagtatttaatattaccacttctaataataattaccacatctcctcaggtaaaaataaataaat
gaataaagccgtgtctcctctgtctcctttacctgtcctaaacctccacaatttaatgggcaatagtccaattttagcatcctcaactgaagtgattat
ccttgggtgactcaattttgattttgtctccatatttgctgtgaagcacttatcgctggggtcgttctgccctattcagagtgcacttgtgctgtcaaac
aatgttgggctgtgacgtttttgtatacatatatacttatacacacatatatatttatatatatgtacttatatatactgttaatcaaaacccataatgaaa
cactgcagtctttatcagtgtgaggccatctacctcatcctctcctgtgcattcagtggcgcttggttgtcatgcaggtggatggtgataatataag
ggtggtgtagagcttccagacaggagatggtgccagataagaagacaggccctttcagggaggtagagaagcccccccaggagtcagcaa
gttagcccatgagctaagaggaagtactgtacctgataccaagtagcatttaccattgctgtactctcagtataacttattcca

>Male scaffold107747 (Contains homologous sequences to the exon 4 of *Dmrt1*)

ctcagtgccatgaaaatgcatttagaaacaggctctggagaggtctttttatcaagttatcatcctctcatctgtgatttctgcagaacacctctttg
agttcatgtcagtgaagaaaccaaccctaactgtaaggaaaataataaattacattacacacaattaatcagctaatgttacctgactttactccat
gtccagtatagcttaatttactggacaaccacaacaagttcaaatagaggatagtatgagaaggtcctatagccttgactctgttctggatgtaac
ctaccacgcccagaaaacctctgaaggagggtgctgaggagatatccagatcagatctgcgggctgtcttttgaaggagcagtggctctactc
tgagcccatccagcctgcagagggaactcatttatgcagcctgtatctgtgaagtcattctttcggtcgctacccaaagctcctcaccacagactt
tccagctcagcttgagtattactgttgatgctgcaccaatctgcctgtcgaacatctcagaacaatgtctattttgtgcattatttgggatactgaaca
tacagacacgactttcagcccaccaataacactgagaaaggtttatgaactgttaatatggcttcacattgacnnnnnnnnnnnnnnnnnnn
nnnnnnnnnnnnnnnnnnnnnnnnnnnnnnnnnnnnnnnnnnnnnnnnnnnnnnnnnnnnnnnnnnnnnnnnnnnnnnnngttggggttactctgtg
tggatccttctctgtggcagaacctgcctttttgagcaactttcaataagacagttttgtttggtgctgtatgtgtcctgaaaactgtaagcttagtgc
actctcaagacccagttcaatctccaatgtatttttcacagcctaatacaagagctgagtgtagcttaaataacagtttataaagctgtcggagaat
tgatggtgtggacaatacgggtgacatattacaggctgacagaaatcaaattccataaaagtgaaattgaaaaccctctgcgaggcggcctcg
ctgctgagtagacagctcgttttaaagttttcagaggactgaaagcagcgtgttgcagctcatttttagaaaaggccacaacccatgaattcaaa
cactactaattctctgttattattttacctctatatttcatcctttcagattaggttgtttaagtattgcctgtgaatatctcatggccagtttggagaactg
tacagtcaaacatgggtgaactgagtcatagtacaatggtattcatattgtatttgaaatttcaaatatagaaaaggaaccttctttaaaaaaaagtt
atatgataaaaaaaacaatcgtacaatgcatgactcggatgttgtctgtgaaatacagcacatgACGACAATTCTTTATATCAG
CCTCCTGTCGAGCCGCAACATGTCCTCTCGGTACTGCATGCATTCCTACTACCCAGCAG
ACACCTACTCGGCTCAGAGACTGGATTCCACCCCCTGTGTGCCAAGTGTCTTCAACCT
GGAGGACAACAACAGCAACAACAACAATGACAACAATGACAACAACTGCTCTGAGG
CCATGGCTGCATGTNNnnnnnnnnnnnnnnnnnnnnnnnnnnnnnnnnnnnnnnnnnnnnnnnnnnnnnnnnnnnnnnn
nnnnnnnnnnnnnnnnnnnnnnnnnnnnnnnnnnnnnnnnnntacacacaaaacactcacacacacttcccaggccccataaagtgttactc
gggctttatccacagtgtagttaagtgcgttcaagggagaat

>Male scaffold68937 (Contains homologous sequences to the 3` UTR of *Dmrt1*)
TTTGATAGGGAAAACATTTTAAATAATTGTAACCATTGTACTGAGCAGGATAATTCATCA
GACCATTTGTTTCTAAATGAGCTCAAACATGTCTTGTCTTTTCTGTACAATAATTCCATG
TCAGTCATCAGGTGACACATACAAATATATCTGCAATAAGCTATTTTTTCTTGGACAATT
TATTGGTGGATGACAAGACACCTTTTGACTTGCAACATTGGAAAGTACTCTGAATTAAA
TACTGTGATTTACCTCAAACAACGATCGGATCAATGGGAAAATCAGATGATTGCGAAGC
TGCTGAAACTGCACACGTTCTTTCTTTTTCTGGTATAGATGAAAGTTAATTAAAGCTCA
GAGCAGGGAACCTGTCTGAGTGTCTCCTCCTGCAGGACAATGTGCAATTCGAGGGTTA
GGGGGGACAAAAAGCCAAACATGAATCATATTTCCCTTTGTTGTGTTGGTGTGTGATGA
GCAACGACTGCTTATTTATGGCGCTGATCCGGCAGCACCGTTTATACCACAGAATTTGC
ATGTCTGTTGTATATTTAACCCCTTCTGGCCAGGAGTTTTGCTGTGCACACGGGTTACTC
TTTACACACTTTGGTACTGCCTCGTAGATACAGCAGAGGATGAGGGGGTGAATCTGTAG
CAGTGATGTGTAGAGCAGGAATATTTGAGTTTATTATCTGATTAAGTCATCTAGAAAATG
AGTCAGCATCTAATTCGAGTCGTTGGTCAGTCATTTAAGGCATTTTCCAAGACAAAAAA
GTAAAACATTTTATAGTTTCAGCATCTCTTTGGATTTGCTGTTTTTTCTTGTCCTGAGTTG
TAGTAAATTGAATATCTTTGAGTTCTGGACTGTTGGGATCTGGCCATTTTTCATGTTTGT
TTTGTGATGATTTACAGACCCAGTAACATTAATCAAACCCCAAATCAAACTAAAGTTGT
TTTTTACTTCATGAACTAATGATAGTAATCCTGTTTTGCCCACTTATGAGTTGTGTTGTTT
AAGTGTTAATTAAAAAACAACACAGATGAGATTTTGCCAATTTCTCTGCATTGTGAAGC
TGACTAGTACATGGCTGTATTGTATATTGCTTGTGTTTTATGGAGATTCATTTCAAACTGC
ATGCTTTGAA

>Male scaffold118347   (Contains homologous sequences to the 3` UTR of *Dmrt1*)

GACTTAATTGGATTTTACAGAAGATTGAGTTTTATTGCCCCGTGCTTTACGAGTTTAGTT
AAGGAAGGCCATTTTATCATGGTTGTCAGGGGTGAGCTGCTGTGCTGCCTTGTCCTTCT
GTAGCTGCTGCTGCCCTGTGTACAGCGCAGGGGAGAAAGTGTCACAGGATTGGTTTTC
CTTGAGCTGCCTCCACCTCCATGAATCATGCCCTCCCATAATAGATAAAATCTGCTCGGA
TCCCACCGTGACACCTCCTGGTGGAGTGATGGCCGCTCGACACAGACGAGAAGTGTG
GAAGAGGACGCCGTAAGATGTTGGTGTATACTTGTGTATACTTTGGATGTAGTTTCATCT
GAGTGATGTAGATGAAATATGTGTGATGAGGTCATCATACAGGATCATACAGGATTTTG
GGAAGCTCTTTATGCCTGTAGGTAGGGCTGAACATGTTCAATAAGACAAAAATTGGTCT
GAAATGTGTACCACTTGAAGTTTAGGCAACATGATCTGCAACTTATTTGCATTCTCTCCT
TTTATTCCTCTTTACCTTTTTTTGGTGAAAATCTGTAACAGCATCCTTAATGCTGAGCCTT
AGGTTAAAGTTTAGTATATACCTTGTTTAAATGTTGAAGATTAGTTAGCAAACCAGTACA
AACTATATGCTCATTCAATAAAGTACGTGACAAGGCCTGTTAATGTTGACAGACATCTT
CTTTGGCTAAATACATCTGATTGAATGATTCAGATATTAAGAAATATTATTCTGTTTTCCA
AGGTTTGATTATTGTGTGTATTTGTTCCAAATATTTATATGAAGAAAGATATGCTTTTCTT
TTTTATGACGTGATCTGTTGTGGGTTCAATACCTCCGCGTTTACTGTGATATATTCAGATT
TTTCGAATAAGAGATCAGAAATATGAGTTTATCGTGATAGATCTACTGGCGGATTTTGGC
ATGTTTGCAATAAATCATCTGATTTCTACAGTTTGTCAGTTCTTCCATCCATCCATCTTCT
TCCGCTTCACCCGGGACCGGGTCGTGGGGGCAGCAGCTTGAGCAGGGATGCCCAGAC
TTTCCTCTCCCCAGACACTCTCTCCAGCTCTTCCGGGCAGACCCCAAGGCGTTCCCAG
GCCAGCCGAGTGACATAGTCTCAATATAAAAGTAAAATTCTCACAGGTAAGAGTGGAG
CTGATTTGAACTATTTTATAATGTTCAGAGAAAACATAATTAATTATTGAGTCTTAAAAG
TATTTTTCACAACATAAGTGGAGAGTCAAGCTACATTATTTCATTTATAGAAAACAACTC
GTTTCAGGAACCTTTTAGAAATGAGACAGTAAAACAGAATAAGGGATAGCTGCTTTAG
ACTCAAGAGAAGTGTGACTTAATCCCAGAAAATAGTTTCCTCCTGTTAAAACAAGCTG
AAATTGGAAATATCCAACTACAGTACAAGTGCCTGTAATTTGGTCACATAAAAATGTGG
TAATCTGAAACTAATCTGTATCGACAATTAAAGCAACAGGGGCACATATTTTGCAGAAC
CCCTAAACAAAATCGAACACTTCAAAATCATACAGCAGTTTTATCAGCATCTTTTATATG
CTGAGATGCTGATTTGTACAATTTTATCCAATATTAAATCATTTTGGCCACTTTCCTGTCC
ACTTCCCTAACAAATAAGATAAATGTCAATCATGTGTTTCATAGGGTTTTGGTCAAGTC
AAGTCAAGTCAAGTCAACTTTATTTGTACA
